# Genome-Wide Identification of Candidate Loci Associated with the Regulation of the Protein, Oil, and Carbohydrate Content in Soybean

**DOI:** 10.3390/plants15060924

**Published:** 2026-03-17

**Authors:** Sreeparna Chowdhury, Byeong Hee Kang, Won-Ho Lee, Seo-Young Shin, Da-Yeon Kim, Woon Ji Kim, Jeong-Ho Baek, Seong-Hoon Kim, Bo-Keun Ha

**Affiliations:** 1 Department of Applied Plant Science, Chonnam National University, Gwangju 61186, Republic of Korea; sreeparna1996@gmail.com (S.C.); rkdqudgml555@naver.com (B.H.K.); dnjsgh201115@naver.com (W.-H.L.); shinsy112345@naver.com (S.-Y.S.); lipi123@naver.com (D.-Y.K.); dnswl007@naver.com (W.J.K.); 2 BK21 Interdisciplinary Program in IT-Bio Convergence System, Chonnam National University, Gwangju 61186, Republic of Korea; 3 Department of Agricultural Biotechnology, National Institute of Agricultural Sciences, Rural Development Administration, Jeonju 54874, Republic of Korea; firstleon@korea.kr; 4 National Agrobiodiversity Center, National Institute of Agricultural Sciences, Rural Development Administration, Jeonju 54874, Republic of Korea

**Keywords:** soybean, genome-wide association study, primary metabolite, protein, high-throughput phenotyping, NIR-GrainSense, candidate gene, haplotype analysis

## Abstract

Soybean (*Glycine max* (L.) Merr.) is a globally important legume valued for the high protein, oil, and carbohydrate content of its seeds. However, it is difficult to simultaneously optimize the content of these three macronutrients due to their negatively correlated metabolic pathways and complex quantitative inheritance patterns. In this study, a genome-wide association study (GWAS) was conducted to elucidate the genetic architecture underlying the seed protein, oil, and carbohydrate content in 328 soybean accessions evaluated over two years using near-infrared spectroscopy. Significant negative correlations (*r* = −0.323 to −0.656, *p* < 0.001) were observed between the three traits, confirming the trade-off in carbon partitioning during seed development. The GWAS identified nine significant loci distributed across chromosomes 4, 6, 8, 9, 10, 16, and 18 as stable quantitative trait loci (QTLs) regulating the three traits. Of these, three pleiotropic loci (*qProOil.4*, *qProOil.16*, and *qOilCarb.6*) were found to be associated with multiple seed compositional traits. Haplotype analysis revealed seven haplotype blocks with distinct phenotypic variation, indicating that they have the potential for use as trait-specific markers in marker-assisted selection. Functional annotation of the stable QTL regions identified 22 putative genes, among which five candidate genes, *Glyma.06g201700*, *Glyma.08g281900*, *Glyma.09g164900*, *Glyma.13g155600*, and *Glyma.16g209800* were likely to be involved in carbon allocation, protein biosynthesis, lipid metabolism, and carbohydrate modification pathways based on their relative expression levels. Overall, this study enhances the understanding of the genetic and molecular mechanism controlling the composition of soybean seed and identifies promising genomic targets for precision breeding programs aimed at improving nutritional quality.

## 1. Introduction

Soybean is a globally significant legume crop due to the composition of its seeds, which are high in protein, oils, and carbohydrate content. Collectively, these three components constitute the vast majority of the dry weight of soybean seeds and define their quality, processing attributes, and economic value [1,2,3]. Protein generally accounts for 35–40% of the dry weight, thus soybean is an important source of plant-based protein for food and animal feed. Similarly, with an average oil content of 18–22%, soybean is also a major source of edible vegetable oil for human consumption and a raw material for biofuels [4]. The carbohydrates in soybean seeds, accounting for ~35% of the dry weight, include dietary fiber, sucrose, and raffinose family oligosaccharides (RFOs) and are closely associated with seed development, processing quality, and feed digestibility [5]. The balance of these three components is thus a key determinant of the market value, processing suitability, and breeding potential of soybean.

Recent studies have focused on the integrated analysis of the protein, oil and carbohydrates in soybean to elucidate their metabolic and biosynthetic interactions during seed development. Carbon partitioning within the developing seed determines the allocation of assimilates into storage proteins, triacylglycerols, and carbohydrates through key metabolic pathways, including sucrose transport, glycolysis, fatty acid biosynthesis, and storage protein synthesis [6,7]. However, these partitions exhibit complex inheritance patterns due to strong genetic and physiological correlations. Competition for carbon substrates during seed filling results in negative correlations between protein and oil levels [1], while carbohydrate levels tend to be negatively associated with protein and have a variable relationship with the oil content depending on the proportions of the different sugars (e.g., sucrose and RFOs) present [8,9]. Consequently, the improvement of one component often leads to a reduction in the others, complicating attempts by breeders to simultaneously improve multiple seed composition traits. Therefore, it is important to understand the genetic and metabolic mechanisms underlying these relationships in order to develop breeding strategies that can optimize overall seed quality.

The phenotypic evaluation of seed composition traits has generally relied on conventional analytical methods such as Kjeldahl assay for protein, Soxhlet extraction for oil, and colorimetric assays for carbohydrates. Although these methods produce accurate measurements, they are labor-intensive, time-consuming, and expensive, making them impractical for large-scale breeding programs. In contrast, high-throughput near-infrared reflectance (NIR) spectroscopy represents a rapid, non-destructive, and cost-effective alternative for estimating seed compositional traits with suitable accuracy [10]. This technology enables the efficient phenotyping of large breeding populations from various environments, thus facilitating large-scale genetic analysis. Therefore, reliable, high-throughput phenotypic data are required for modern genomic studies.

Genome-wide association studies (GWAS) have become widely used to the genetic architecture of complex quantitative traits such as the protein, oil, and carbohydrate contents [11,12,13]. By exploiting natural allelic variation within diverse germplasm panels and integrating high-density single nucleotide polymorphism (SNP) genotyping, GWAS can be used for the high-resolution mapping of loci associated with complex quantitative traits [13]. Over the past decade, various studies using both natural and bi-parental populations have identified numerous QTLs controlling the protein and oil content [14], through relatively few have explored the genetic basis for carbohydrate levels [15]. In particular, more than 300 QTLs for oils and 240 QTLs for proteins have been reported in soybean to date [13,16], compared with only 53 QTLs associated with sucrose, starch, RFOs, and other monosaccharides [17].

Functional studies have also identified several key genes regulating these traits. Members of the *SWEET* gene family (*GmSWEET10a*, *GmSWEET10b*, and *GmSWEET15*) play significant roles in coordinating the accumulation of both proteins and oils [18]. To date, more than 30 functional genes influencing the oil content have been characterized, including important transcription factors such as *GmbZIP123*, *GmDOF4*, *GmDOF11*, and *GmMYB73* [19]. The overexpression of *GmWRI1* and *GmOLEO1* has also been shown to significantly increase the total oil content, while *GA20OX* and *NFYA* have been implicated in the regulation of both seed weight and oil accumulation [20]. In addition, genes such as *RS3* (*Glyma.05g003900*), *RS2* (*Glyma.06g179200*), and *MIPS1* (*Glyma.11g238800*) have been employed in marker-assisted breeding programs for carbohydrate improvement [21], while *Glyma.10g154400* (associated with stachyose content) and *Glyma.11g136200* (associated with sucrose content) were recently identified in cultivated soybean seeds [15]. A variant of the DGAT1 gene encoding type 1 diacylglycerol acyltransferase has also been reported to enhance oil accumulation while reducing the soluble carbohydrate content in soybean [22].

Despite these advances, significant gaps remain in the understanding of the genetic basis for these traits. Many QTLs exhibit strong environmental dependence, which complicates their validation and limits their utility in breeding programs. In addition, the molecular mechanisms linking genetic variation to the metabolic trade-offs among proteins, oils, and carbohydrates remain largely unresolved. To address these gaps, this study utilized multi-environment data from 328 soybean accessions obtained using NIR-based phenotyping, combined with high-density 180K Axiom^®^ SoySNP array (Affymetrix, Thermo Fisher Scientific, Santa Clara, CA, USA), to conduct a GWAS for protein, oil and carbohydrate content in soybean seeds. This integrative approach was used to identify key QTLs and candidate genes regulating the accumulation and metabolism of seed compositional traits, thus providing molecular insights into the complex metabolic network governing these traits.

## 2. Results

### 2.1. Phenotypic Evaluation of Seed Compositional Traits in Soybean Accessions

The protein, oil, and carbohydrate content of seeds (on a dry weight basis) was evaluated in 328 soybean accessions across two consecutive years (2020 and 2021). These three traits exhibited wide phenotypic variation between accessions, indicating the presence of considerable genetic diversity within the population (Table 1). The mean protein, oil, and carbohydrate content across the two years ranged from 33.63 to 49.88%, 15.27 to 28.83%, and 29.58 to 40.43%, respectively. The oil content exhibited the highest coefficient of variation (CV = 10.75%), followed by carbohydrate content (CV = 5.44%) and protein content (CV = 5.23%). All three traits had a high broad-sense heritability (H^2^ ≥ 0.7), suggesting that genetic factors were the dominant contributors to phenotypic variation, with relatively minor environmental effects.

**Figure 1:**
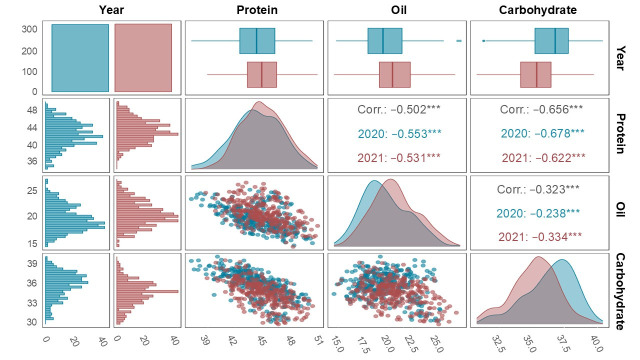
Correlation matrix illustrating pairwise relationships among protein, oil, and carbohydrate levels of soybean seeds in 2020 (blue) and 2021 (red). Diagonal panels present histograms and density plots, while the (**lower**) panels show scatter plots with fitted trends. The (**upper**) panels present Pearson’s correlation coefficients between traits, with *** indicating significance at *p* < 0.001. Values shown in black denote the average correlation coefficients across the two years.

Pairwise correlation analysis revealed significant negative relationships between the three seed composition traits (Figure 1). Protein content was strongly and negatively correlated with the oil (*r* = −0.502, *p* < 0.001) and carbohydrate (*r* = −0.656, *p* < 0.001) content, whereas a weak but significant negative correlation was observed between the oil and carbohydrate content (*r* = −0.323, *p* < 0.001). These results indicate that an increase in one trait often occurs at the expense of another, reflecting metabolic trade-offs and inverse regulatory relationships among the biosynthetic pathways controlling seed composition in mature soybean seeds.

### 2.2. Genome-Wide SNP Distribution and Detection of Significant SNPs from the GWAS

**Figure 2:**
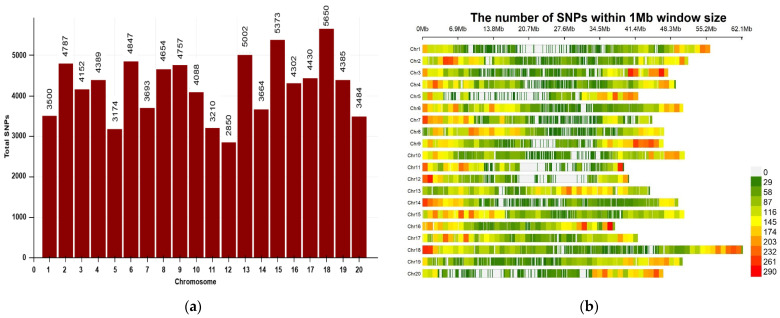
Distribution of genome-wide SNPs from the GWAS population across 20 soybean chromosomes. (**a**) Bar plot demonstrating the number of SNPs per chromosome. (**b**) Heatmap representing the SNP density within a window size of 1 Mb. The color scale illustrates an SNP density ranging from green (lower density) to red (higher density). The *x*-axis indicates the physical position along each chromosome, while the *y*-axis corresponds to the 20 soybean chromosomes.

A total of 84,391 high-quality SNPs, derived from the filtered 180K Axiom^®^ SoyaSNP array (Affymetrix, Thermo Fisher Scientific, Santa Clara, CA, USA), were used for genome-wide association analysis of the protein, oil, and carbohydrate content (Supplementary Table S1; Figure 2). The SNPs were unevenly distributed across the 20 soybean chromosomes. Chr. 18 had the highest number of SNPs (5650) followed by Chr. 15 (5373), while Chr. 12 (2850) and Chr. 05 (3174) had the lowest number of SNPs per chromosome. In terms of SNP density, Chr. 16 (115.10 SNPs Mb^−1^) and Chr. 13 (113.23 SNPs Mb^−1^) had the highest marker density, while Chr. 01 (62.64 SNPs Mb^−1^) and Chr. 12 (71.10 SNPs Mb^−1^) had the lowest. This indicates moderate variation in marker coverage between the chromosomes.

**Figure 3:**
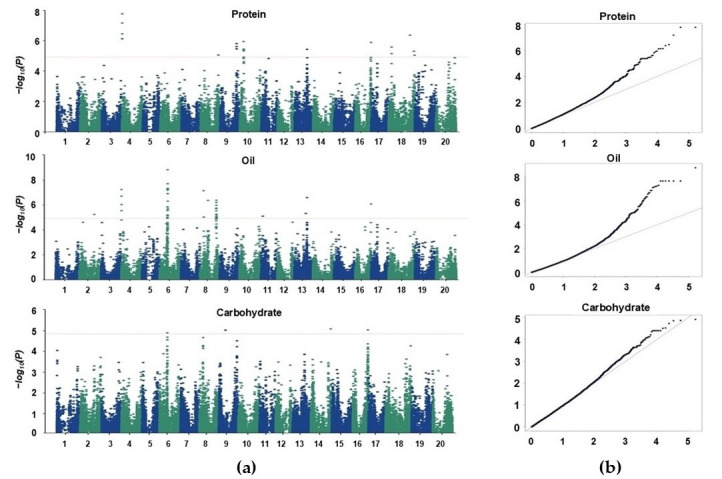
GWAS signals for seed compositional traits in soybean accessions. (**a**) Manhattan plots and (**b**) Q–Q plots for the protein, oil and carbohydrate contents. In the Manhattan plots, the *x*-axis represents chromosome number and the *y*-axis shows the −log_10_(*P*) values. The horizontal red line indicates the genome-wide significance threshold (Bonferroni-adjusted).

The phenotypic data collected during 2020 and 2021 growing seasons were merged prior to GWAS analysis to incorporate observations from both years, thereby increasing the statistical power and improving the robustness of significant SNP detection across seasons. Consecutively, the GWAS was conducted by integrating genotypic and phenotypic datasets to detect loci associated with the three seed compositional traits (Figure 3). The Q–Q plots showed that the observed *p*-value distribution closely followed the expected null distribution with deviation only at the tail, suggesting true SNP–trait associations. The genomic inflation factor (λ) ranged from 0.82–0.97 across traits, indicating minimal inflation and confirming that the MLM model effectively controlled for population structure and relatedness. Distinct association signals were observed across multiple chromosomes, reflecting the polygenic nature of these quantitative traits. In total, 101 SNPs distributed over 10 chromosomes were identified as significantly associated with the target traits (37, 61, and 4 SNPs with protein, oil, and carbohydrate content, respectively). Of these, nine major peak SNPs, including loci overlapping among traits (pleiotropic) and those exhibiting strong linkage disequilibrium (LD) with neighboring markers, were defined as stable SNPs and selected for downstream analysis (Table 2). Subsequently, the R^2^ values for the SNPs represent the coefficient of the fitted model for each trait rather than the proportion of phenotypic variance explained by individual SNPs.

Two SNPs, AX-90363747 on Chr. 04 (337,536 bp) and AX-90496790 on Chr. 16 (36,857,004 bp), had significant associations with both the protein and oil content. In addition, AX-90471463 on Chr. 06 (18,670,261 bp) exhibited a significant association with both the oil and carbohydrate content. The remaining six significant SNPs were distributed across chromosomes 8, 9, 10, 13, and 18, each displaying trait-specific effects. Collectively, these results highlight the complex but partially overlapping genetic basis for the regulation of seed compositional traits in soybean.

### 2.3. Allelic Effects of Significant SNPs Associated with Seed Compositional Traits

The allelic effects of the selected stable SNPs (referred to as QTLs) were evaluated by comparing the mean values of each trait among soybean accessions carrying different alleles. This analysis was used to determine both the statistical significance and the direction of the allelic effects in relation to their corresponding phenotypic traits (Table 3). A single allelic effect was represented by a mean difference between the accessions carrying the reference and alternate alleles. A positive allelic effect indicated that the reference allele contributed positively to the trait value, whereas a negative effect indicated that the contribution of the alternate allele was favorable.

For protein content, most of the SNPs had negative allelic effects, indicating that the alternate alleles were more favorable for protein enhancement. The only exception was AX-90468327 on Chr. 10, which had a positive effect, with higher protein content found in accessions carrying the reference allele (TT). In contrast, all of the SNPs associated with the oil content had positive effects, suggesting that the reference alleles enhanced oil accumulation. Of these, AX-90435033 on Chr. 8 exhibited the largest allelic effect (2.19%), representing the maximum difference in the oil content between allelic groups. For carbohydrate content, AX-90471463 on Chr. 6 had a negative effect, indicating that accessions carrying the reference allele (TT) had a lower carbohydrate content compared with those with the alternate allele [23]. Interestingly, this same SNP (AX-90471463) had a positive effect on oil content, which was consistent with the inverse relationship between these two traits. Similarly, the overlapping SNPs AX-90363747 (Chr. 04) and AX-90496790 (Chr. 16) had opposite allelic effects for the protein and oil content. Together, these findings support the negative correlations observed among the protein, oil, and carbohydrate content (Figure 1).

### 2.4. Haplotype Analysis of Stable QTLs Associated with Seed Compositional Traits

**Figure 4:**
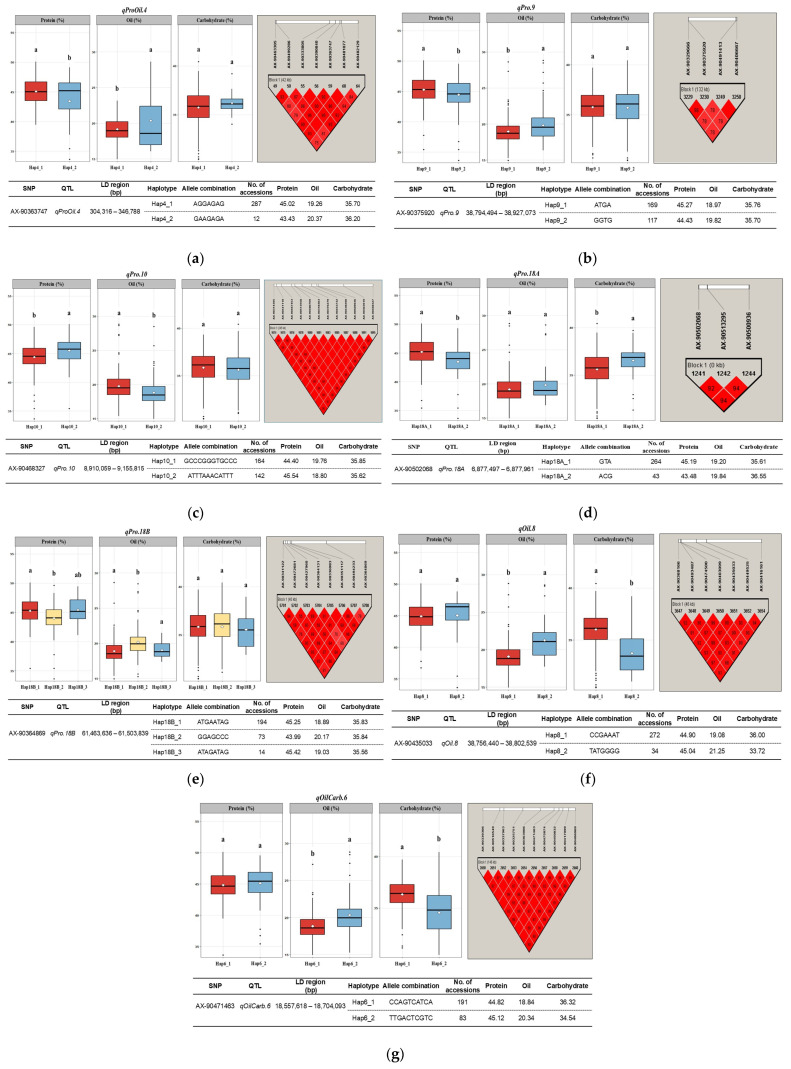
Haplotype analysis of stable QTLs associated with seed compositional traits. Each panel (**a**–**g**) presents (i) boxplots showing phenotypic variation in seed compositional traits between different haplotypes, (ii) the corresponding LD block (*r*^2^ ≥ 0.8), and (iii) a haplotype summary table. Statistical significance was determined using Welch’s *t*-tests (two groups) or Welch’s ANOVA + Games–Howell test (≥3 groups) at *p* < 0.05. Different letters (a and b) indicate significantly different groups, while identical letters denote non-significant differences.

To further examine the combined influence of multiple SNPs, haplotype analysis was conducted within LD blocks, including each significant locus (Figure 4). All of the identified QTLs except *qProOil.16* and *qOil.13* showed a strong LD association with adjacent SNPs for seed compositional traits, as represented by their haplotype blocks.

Within *qProOil.4*, two major haplotypes were detected: Hap4_1 (AGGAGAG) and Hap4_2 (GAAGAGA). Accessions carrying Hap4_1 had higher protein but lower oil content, while those with Hap4_2 exhibited the opposite trend. These differences were primarily governed by allelic substitution at the 5^th^ position (G/A) of the haplotype, corresponding to the significant SNP (AX-90363747) site detected in the GWAS. This suggests that this locus represents a strong associated marker influencing the trait or was closely linked to the true causal variant within the LD block.

Similarly, the *qOilCarb.6* locus contained two haplotypes, Hap6_1 (CCAGTCATCA) and Hap6_2 (TTGACTCGTC), which exhibited contrasting associations between oil and carbohydrate content. Accessions carrying Hap6_1 had lower oil but higher carbohydrate content, whereas Hap6_2 was associated with higher oil and lower carbohydrate content. These contrasting effects were governed by C/T substitutions at the 1st, 2nd, 6th, and 9th positions within the haplotype sequence.

Notably, *qPro.9* and *qPro.18B* also exhibited significant haplotype–trait differences similar to the patterns observed above, while *qPro.10* showed the opposite trend (Figure 4). Additionally, *qOil.8* (Hap8_1 and Hap8_2) and *qPro.18A* (Hap18A_1 and Hap18A_2) had significant haplotype-trait associations, with *qOil.8* influencing the oil and carbohydrate content, and *qPro.18A* affecting the protein and carbohydrate content.

### 2.5. Identification of Candidate Genes Associated with the Protein, Oil and Carbohydrate Contents

**Figure 5:**
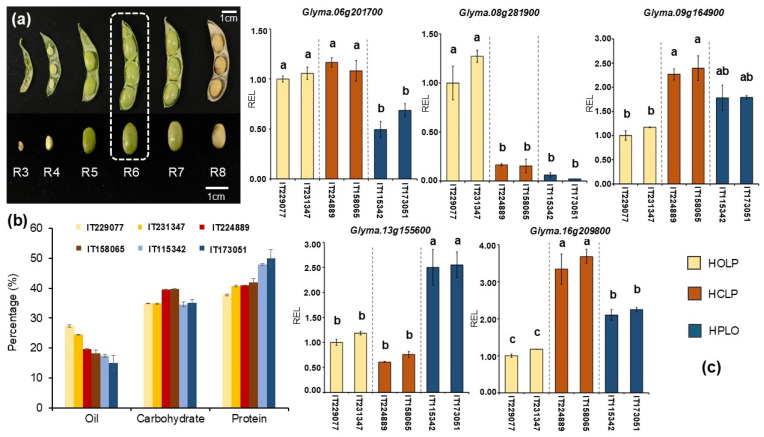
Comparative phenotypic variation and gene expression for high oil–low protein, high carbohydrate–low protein and high protein–low oil soybean accessions. (**a**) Seed development stages of soybean. Seeds at the R6 stage (highlighted with a dashed box) were used for analysis. (**b**) Average phenotypic values of all six accessions for protein, oil, and carbohydrates. The error bars represent the standard deviation across the two years for each accession. (**c**) qRT-PCR analysis showing the relative expression level (REL) of five candidate genes, *Glyma.06g201700*, *Glyma.08g281900*, *Glyma.09g164900*, *Glyma.13g155600*, and *Glyma.16g209800* associated with seed composition in soybean. Differently colored bars on the *x*-axis represent different accessions, and the labels HOLP, HCLP, and HPLO indicate high oil–low protein, high carbohydrate–low protein, and high protein–low oil accessions, respectively, while the *y*-axis presents the REL of each gene. IT1229077 was considered the control. Error bars indicate the standard error, with different letters indicating statistically significant differences (Duncan’s test, *p* < 0.05).

The genomic regions surrounding the nine stable QTLs were investigated to identify putative candidate genes associated with seed compositional traits. Each QTL interval was examined within the haplotype-based LD block or a ±153 kb physical window around the significant SNP to capture proximal gene candidates. In total, 125 genes were detected within these intervals, among which 22 putative genes distributed across eight QTLs were prioritized based on previous studies, gene functional annotation, and RNA-seq expression data (Table 1; Supplementary Figure S1). Overall, the identified putative genes were functionally associated with enzymes and metabolites of the TCA cycle, protein synthesis regulation, TAG metabolism, nutrient assimilation during seed development, and seed weight determination. Five of these candidate genes (*Glyma.06g201700*, *Glyma.08g281900*, *Glyma.09g164900*, *Glyma.13g155600*, and *Glyma.16g209800*) exhibited differential expression for distinct accessions among the groups (Figure 5). Specifically, *Glyma.06g201700* showed 1.5~2-fold decrease in expression for high protein–low oil accessions, while *Glyma.08g281900* exhibited 5~10-fold downregulation in high carbohydrates and high protein accessions, respectively. In contrast, *Glyma.09g164900* and *Glyma.16g209800* showed approximately 2.2~3.6-fold increased expression in high carbohydrate accessions and 1.7~2.3-fold increase in high protein accessions, respectively. Furthermore, the gene *Glyma.13g155600* exhibited increased transcript levels (2.4~2.6-fold) for the protein-rich accessions IT115342 and IT173051, when compared with the others. The qRT-PCR expression patterns were consistent with the phenotypic contrast among the groups and provide preliminary support for prioritizing these genes within the detected QTLs as candidate regulators of seed protein, oil, and carbohydrates in soybean.

## 3. Discussion

Improving traits associated with seed quality is a fundamental goal in soybean breeding because these traits directly influence nutritional value and market competitiveness. Protein, oil, and carbohydrates are the major macronutrients in soybean seeds that determine both their dietary quality and processing suitability. Therefore, the selection of accessions and the development of new cultivars must consider specific end-use objectives to enable targeted breeding strategies that enhance the overall seed composition. However, these traits are inherently complex and polygenic, regulated by numerous loci with small individual effects and strongly influenced by environmental conditions.

To overcome these challenges, advanced molecular breeding methods have been integrated with high-throughput phenotyping approaches. It has been shown that GWAS combined with NIR spectroscopy-based phenotyping, provide an effective framework for the large-scale analysis of complex quantitative traits in soybean [21]. NIR spectroscopy uses the wavelength-specific absorption and reflectance properties of molecular bonds, to rapidly and accurately quantify seed protein, oil and carbohydrate content [10]. In the present study, NIR-based phenotyping combined with the high-density 180K Axiom SoySNP array across 328 accessions enabled the precise identification of major QTLs and candidate genes regulating the accumulation of seed protein, oil, and carbohydrates.

The evaluated traits displayed wide phenotypic variability, reflecting substantial genetic diversity within the population, which is required for reliable GWAS analysis (Table 1). The protein and carbohydrate content had relatively low CVs (5–6%), indicating their genetic and environmental stability, while the oil content exhibited a higher CV (≥10%), suggesting greater potential for selection in breeding programs [13,24]. High broad-sense heritability values (*H*^2^ = 0.7–0.96) also indicated that most of the phenotypic variation between accessions was genetically determined, supporting the feasibility of selection based on allelic variation [13,25]. Although the trials were conducted at a single location, phenotypic evaluation across two independent growing seasons allowed partial assessment of environmental variability. The consistently high heritability values suggests that genetic effects predominated over environmental influences, implying that the loci identified in this study are likely to represent relatively stable genomic regions controlling seed composition under the experimental conditions. In addition, significant negative correlations among the three traits (r = −0.323 to −0.656) confirmed the metabolic trade-off in carbon partitioning between protein, oil, and carbohydrate biosynthesis during seed development [9,19].

GWAS analysis identified three loci (*qProOil.4*, *qProOil.16*, and *qOilCarb.6*) showing pleiotropic effects, with simultaneous association with multiple traits (Table 2). Specifically, SNP AX-90363747 on Chr. 4 (0.33 Mb) which was significantly co-associated with both the protein and oil content, was located in close proximity to the previously reported *qPro4* QTL (0.56 Mb), which controls the seed protein content [26]. Similarly, AX-90496790 on Chr. 16 (36.85 Mb) corresponded to previously identified QTLs, including *qPro-6* (36.10 Mb) for protein content [27] and *Seed linoleic 1-g19* and *Seed oleic 1-g23* (37.33 Mb) for seed oil composition [28]. Additionally, AX-90471463 on Chr. 6 (18.67 Mb) had a pleiotropic association for oil and carbohydrate content, which is consistent with a previous reported SNP (Gm06_19321023, 19.32 Mb) linked to fructose levels [29]. Comprehensive haplotype analysis of the stable QTLs elucidated their multi-allelic effects and revealed superior haplotype combinations associated with a more favorable seed composition. Consistent with previous studies identifying haplotypes affecting oil [30], protein [7,31], and carbohydrate components [32], seven major haplotype blocks (*qProOil.4*, *qPro.9*, *qPro.10*, *qPro.18A*, *qPro.18B*, *qOil.8*, and *qOilCarb.6*) were found to regulate variation in seed composition (Figure 4). Accessions carrying favorable haplotypes displayed significant phenotypic advantages, suggesting that these multi-allelic combinations were suitable as trait-specific markers for marker-assisted selection in soybean breeding.

Functional annotation of the putative genes within the identified QTL regions (Table 4) suggested that several loci may influence seed composition through genes involved in carbon assimilation, energy metabolism, protein turnover, and biosynthetic regulation. For instance, genes associated with carbon metabolism and carbohydrate processing, including *Glyma.04g003900* and *Glyma.16g209800* encoding galactosidases may regulate reported as implicated in carbon flux modulation and carbohydrate degradation during seed development in soybeans and common beans, thereby influencing the portioning of storage compounds [33,34]. Likewise, genes related to cellular energy metabolism, *Glyma.09g164900* (succinyl-CoA synthetase) and *Glyma.10g079500* (serine/threonine-specific protein phosphatase), participate in the TCA cycle and metabolic signaling, influencing the energy and assimilates supply. Previous GWAS and QTL studies have also highlighted the importance of these genes in regulating seed protein and oil content in soybean [34,35].

In addition, several genes identified are involved in post-transcriptional and protein regulatory processes, such as *Glyma.04g004000* (HECT domain ubiquitin-protein ligase), *Glyma.06g201700* (ATP-dependent RNA helicase) and *Glyma.06g202000* (dentin sialophosphoprotein), and translation-related genes like *Glyma.16g208500/208600* (translation initiation factor 6 eIF-6). These genes may contribute to RNA processing and translation mechanisms regulating metabolic pathways during soybean seed growth and development stages. Similar regulatory mechanisms have been reported in previous GWAS studies investigating soybean seed compositional traits [46,47]. Likewise, storage protein gene, *Glyma.16g208900*, which encodes for Bowman–Birk serine protease inhibitor, represents one of the major seed protein inhibitors that account for approximately 6% of total seed proteins, alongside Kunitz trypsin inhibitors [38]. Moreover, genes such as those associated with transcriptional regulation and signaling, including *Glyma.08g281900* (AP2 domain) and *Glyma.18g072800* (BTB/POZ-domain protein), are possibly known to regulate transcriptional networks (*LEC1*, *LEC2*, and *FUS3*) associated with fatty-acid biosynthesis, glycolysis, and starch metabolism. Consistent with previous studies, the transcriptional regulators may implicated the coordinated accumulation of oil, protein, and carbohydrates in soybean seeds [7].

In the qRT-PCR analysis, five candidate genes were found to be differentially expressed between contrasting accessions with high oil, protein, and carbohydrate content (Figure 5). Notably, *Glyma.06g201700*, which encodes for ATP-dependent RNA helicase, exhibited stable expression levels in high oil–low protein and high carbohydrate–low protein accessions. This expression pattern supports previous reports suggesting its potential role in directing the metabolic balance during the seed development stages [47,49]. The gene *Glyma.08g281900*, which encodes for the AP2 domain, exhibited a higher REL for high oil–low protein accessions, while its expression was negligible for high carbohydrates and high protein accessions. This was consistent with a previous study reporting that AP2 domain-containing proteins, particularly WRINKLED1 (WRI1), regulate fatty acid and oil biosynthesis in soybeans [24,50]. *Glyma.09g164900* encoding for succinyl synthetase exhibited higher expression in high carbohydrates and high protein accessions, supporting the earlier findings of its contribution to the TCA cycle by controlling the carbon flux, leading to the accumulation of proteins, oils, and carbohydrates in seeds [34]. In addition, gene *Glyma.13g155600*, which encodes alpha/beta hydrolase showed higher REL in high protein–low oil accessions compared with the other grouped accessions. This is consistent with previous studies, indicating its involvement in amino acid metabolism or nitrogen assimilation and thus contributing to enhanced protein accumulation [26]. Similarly, *Glyma.16g209800* (alpha-galactosidase) was upregulated in high carbohydrate–low protein accessions, while its expression was more moderate in high protein accessions and lower in high oil accessions. This observation supports earlier reports of alpha-galactosidase participating in oligosaccharide turnover and carbohydrate metabolism, indicating its potential role in promoting carbohydrate accumulation during seed maturation [40].

## 4. Materials and Methods

### 4.1. Planting Materials and Phenotyping Evaluation

This study utilized 328 soybean accessions from the diverse GWAS panel, including 97 accessions each from China and Japan, 74 accessions from Republic of Korea, and 60 accessions form North Korea. All accessions were sown (June), cultivated and harvested (November) during 2020 and 2021 at the research station of the Rural Development Administration (RDA), Jeonju, Republic of Korea (35°49′ N, 127°09′ E). The experimental site is located on a temperate climatic zone with an average growing season temperature of approximately 15–27 °C and average precipitation of 64–78 mm. The soil at the site is classified as sandy loam soil with good drainage properties and rainfed irrigation practices, suitable for soybean cultivation. The planting of each accession was done in a two-row layout with the spacing of 0.3 m × 1.5 m (plant-to-row), in two replications, mentioned in detailed by [51]. Harvested seeds from each accession were oven-dried at 40 °C for 24 h to a final moisture content of ≤13%, then stored under controlled conditions for subsequent phenotypic analysis.

To determine the protein, oil, and carbohydrate content, the dried seeds were quantitatively analyzed using an NIR GrainSense Go Analyzer (Grainsense Ltd., Oulu, Finland). This instrument utilizes near-infrared spectroscopy with built-in pre-validated calibration models using reference chemical methods enabling rapid determination of a seed’s compositional traits [10]. The measurements were conducted following the manufacturer’s recommended protocol and each accession was examined for three technical replicates to ensure precision and to minimize error. The arithmetic mean of the three measurements was calculated to obtain a single representative phenotypic value, which was then employed for statistical and GWAS analyses.

### 4.2. SNP Genotyping and the GWAS Analysis

The 180K Axiom^®^ SoyaSNP array (Affymetrix, Thermo Fisher Scientific, Santa Clara, CA, USA) developed by [52] was employed to genotype the 328 soybean accessions. The SNP calling, population structure analysis, and LD were previously estimated by [53]. The same genotypic dataset, comprising 84,391 high-quality SNPs with minor allele frequency > 0.05 after filtration and imputation, was used for the GWAS analysis of the protein, oil and carbohydrate contents. This combination of panel size and marker density provides adequate statistical power to detect loci with moderate genetic effects in soybean.

The GWAS was conducted using the QTLmaxV4 platform (QTLmax Global, Houston, TX, USA), a bioinformatic tool designed for comprehensive genomic data analysis. A mixed linear model (MLM) was employed to evaluate the associations between the SNPs and the three seed composition traits. The standard Q + K version of the MLM [54] was used to minimize false positives while maintaining statistical power. This model incorporates both the Q matrix (population stratification) and the K matrix (kinship) to correct for confounding effects arising from population structure and relatedness [55]. Population structure was previously characterized using principal component analysis (PCA), which revealed the presence of distinct subpopulations within the GWAS panel corresponding to the geographic origins of the accessions [51]. Bonferroni correction (*p* = 1/total number of SNPs after imputation) was used to determine the genome-wide significance threshold (−log_10_(*P*)) to control for multiple testing. The GWAS results were visualized as Manhattan plots and Q–Q plots using QTLmaxV4 and its visualization tool QWorkbench. In addition, the LD decay analysis revealed an initial value of *r*^2^ as 0.45, which declined to half of its maximum to 0.22 at an approximate physical distance of 153 kb (Supplementary Figure S1). Thus, the genomic window of ±153 kb flanking each significant SNP was defined as the putative QTL region for loci consistently detected across years and traits.

### 4.3. Estimation of LD Regions and Haplotype Analysis

The LD heatmaps for significant SNPs identified from the GWAS were generated using Haploview 4.2 [56]. Haplotype blocks were defined as genomic regions containing adjacent SNPs with an *r*^2^ ≥ 0.8 within a physical window of ±153 kb, following the confidence interval algorithm described by [57]. Single-allelic and multi-allelic effects were analyzed to identify favorable alleles and SNP–trait associations governing the seed protein, oil and carbohydrate content. Significant SNPs forming clear haplotype blocks were used to classify accessions based on their haplotype alleles, and haplotype–trait associations were assessed using mean phenotypic values. Haplotypes were designated using the format Hap18A_1, where *18* denotes the chromosome number, *A* represents the order of significant SNPs on the same chromosome, and *1* indicates the haplotype allele number.

### 4.4. Functional Annotation of Putative Genes

Putative genes located within the LD region (±153 kb) or corresponding haplotype block of each significant SNP were identified using the soybean reference genome Wm82.a2.v2, obtained from SoyBase (https://www.soybase.org/, accessed on 19 September 2025). Gene identification and selection were guided by LD-based gene annotation, tissue-specific expression profiles (Supplementary Figure S2), and previously reported trait-related studies to elucidate potential biological functions associated with the seed protein, oil, and carbohydrate content. QTL or LD regions were designated following the convention *qProOil.4*, where *q* indicates a QTL, *ProOil* represents the protein–oil trait group, and *4* specifies the chromosome number.

### 4.5. Gene Expression Analysis

The qRT-PCR analysis was carried out on three contrasting soybean groups, each comprising two accessions: high oil–low protein (IT229077 and IT231347), high carbohydrate–low protein (IT224889 and IT158065), and high protein–low oil (IT115342 and IT173051), which displayed clear differences in phenotypic values (Supplementary Table S2; Figure 5), to evaluate the expression patterns of candidate genes. The soybean plants from each accession were grown at the Experimental Station of Chonnam National University, Gwangju, Republic of Korea, during the 2024 growing season. Fresh seeds were harvested at the full seed (R6) stage with two biological replicates. Total RNA was isolated utilizing a TaKaRa MiniBEST Plant RNA extraction kit (Catalog no. 9769; TaKaRa Bio Inc., Shiga, Japan), and first-strand cDNA was synthesized using the LaboPass cDNA Synthesis Kit (Comso Gene Tech Co. Ltd., Seoul, Republic of Korea). The gene-specific primers (Supplementary Table S3) were designed in NCBI Primer-BLAST (https://www.ncbi.nlm.nih.gov/tools/primer-blast/, accessed on 20 September 2025) and produced by Macrogen (Seoul, Republic of Korea). The qRT-PCR reactions were performed using Power SYBR Green Master Mix on an ABI StepOne Real-Time PCR system (Thermo Fisher Scientific, Waltham, MA, USA) with two biological replicates and three technical replicates per accession. The thermal cycling conditions consisted of an initial denaturation at 95 °C for 10 min, followed by cycles of 95 °C for 15 s and 60 °C for 1 min. Relative gene expression levels were estimated using the 2^−ΔΔCt^ method [58], with IT229077 (high oil–low protein) accession used as a control to normalize 2^−ΔΔCt^ for comparing expression levels of genes among the selected accessions, while the housekeeping gene *GmActin11* (*Glyma.18g290800*) was considered as internal reference gene.

### 4.6. Statistical Analysis

Statistical analyses were conducted using Microsoft Excel 2019, RStudio v2024.09.1 + 394 (Posit Software, Boston, MA, USA), and IBM SPSS Statistics 29.0.2.0 (IBM Corp., Armonk, NY, USA). Descriptive statistics for the protein, oil and carbohydrate content were computed in Excel. Correlation analysis was conducted using the R packages ‘*GGally*’ [59] and ‘*ggplot2*’ [60], while broad-sense heritability (*H*^2^) was estimated using the ‘*Variability*’ package [61]. Post-hoc comparisons among two or more independent haplotype groups of haplotypes were conducted using Welch’s *t*-tests. For qRT-PCR data, Duncan’s multiple range tests were used to assess differences in the relative expression levels among accessions.

## 5. Conclusions

This study conducted GWAS analysis on 328 soybean accessions to investigate the genetic basis for the protein, oil, and carbohydrate content in soybean seeds. Nine significant SNPs were identified as stable QTLs associated with these traits, among which *qProOil.4*, *qProOil.16*, and *qOilCarb.6* exhibited pleiotropic effects. Allelic and haplotype analyses revealed multiple favorable variants within seven major haplotype blocks that effectively distinguished accessions with higher and lower trait values for seed compositions. Integration of GWAS results with gene expression profiling enabled the identification of five promising candidate genes, *Glyma.06g201700*, *Glyma.08g281900*, *Glyma.09g164900*, *Glyma.13g155600*, and *Glyma.16g209800* showing differential expression patterns among the contrasting accessions for protein, oil and carbohydrates. Notably, *Glyma.06g201700*, *Glyma.08g281900*, and *Glyma.09g164900* were co-localized within the detected haplotype region Hap6, Hap 8, and Hap9, respectively, highlighting their potential as candidate targets for marker-assisted selection. These haplotypes may serve as valuable resources for the development of molecular markers to screen breeding population for favorable alleles, thereby facilitating precision breeding for improving soybean cultivars. Nevertheless, the functional roles of the detected candidate genes require further experimental validation. Future studies employing functional genomic approaches, such as CRISPR/Cas9-mediated gene knockout or overexpression analyses, will be necessary to confirm their accurate involvement in soybean seed composition regulation. Collectively, these findings provide new understandings into the genetic architecture of soybean seed composition and establish a foundation for improving nutritional quality through advanced breeding strategies.

## Figures and Tables

**Table 1:** Summary statistics for the seed protein, oil, and carbohydrate contents across two years in 328 soybean accessions.

Trait	Year	Range	Mean ± SE	SD	CV%	Kurtosis	Skewness	H^2^
Protein (%)	2020	34.17–50.10	44.67 ± 0.14	2.58	5.78	1.25	–0.72	0.88
	2021	33.10–50.70	45.25 ± 0.13	2.42	5.35	3.07	–0.87	
	Mean	33.63–49.88	44.96 ± 0.13	2.35	5.23	2.66	–0.91	
Oil (%)	2020	15.15–29.37	19.07 ± 0.12	2.15	11.27	3.13	1.31	0.96
	2021	15.00–29.77	19.67 ± 0.11	2.17	11.20	3.87	1.34	
	Mean	15.27–28.83	19.34 ± 0.10	2.08	10.75	3.98	1.42	
Carbohydrate (%)	2020	29.33–40.53	36.24 ± 0.12	2.17	5.99	0.27	–0.77	0.7
	2021	29.50–41.50	35.08 ± 0.11	1.95	5.56	0.27	–0.23	
	Mean	29.58–40.43	35.66 ± 0.11	1.94	5.44	0.25	–0.62	

SE, standard error; SD, standard deviation; CV, coefficient of variation; H^2^, broad-sense heritability.

**Table 2:** Genome-wide significant SNPs associated with the protein, oil and carbohydrate contents.

Trait	SNP ID	Chr.	Ref. Allele ^1^	Alt. Allele ^2^	Position (bp)	−log_10_(*P*)	MAF ^3^	Model R^2^ (MLM)
Protein	AX-90363747	4	A	G	337,536	7.78	0.10	0.59
	AX-90375920	9	G	T	38,794,608	5.81	0.41	0.59
	AX-90468327	10	T	C	9,155,815	5.46	0.47	0.59
	AX-90496790	16	A	G	36,857,004	5.90	0.10	0.59
	AX-90502068	18	A	G	6,877,497	5.27	0.18	0.59
	AX-90364869	18	C	G	61,503,839	5.05	0.27	0.59
Oil	AX-90363747	4	A	G	337,536	6.69	0.10	0.75
	AX-90471463	6	T	C	18,670,261	8.80	0.32	0.75
	AX-90435033	8	G	A	38,766,325	6.20	0.16	0.75
	AX-90381095	13	T	C	27,108,594	5.31	0.15	0.77
	AX-90496790	16	A	G	36,857,004	6.07	0.10	0.75
Carbohydrate	AX-90471463	6	T	C	18,670,261	4.92	0.32	0.66

^1^ Reference allele; ^2^ alternate allele; ^3^ minor allele frequency.

**Table 3:** Allelic effects of nine significant SNPs associated with the protein, oil, and carbohydrate content in soybean.

Trait	SNP ID	QTL	Chr.	Allele	No. of Accession	Mean Value	Allelic Effect
Protein	AX-90363747	*qProOil.4*	4	**A**	20	43.65	−1.43
				G	287	45.07	
	AX-90375920	*qPro.9*	9	**G**	126	44.50	−0.77
				T	179	45.27	
	AX-90468327	*qPro.10*	10	**T**	143	45.54	1.14
				C	164	44.40	
	AX-90496790	*qProOil.16*	16	**A**	21	42.93	−2.15
				G	289	45.08	
	AX-90502068	*qPro.18A*	18	**A**	46	43.46	−1.73
				G	264	45.19	
	AX-90364869	*qPro.18B*	18	**C**	84	43.94	−1.38
				G	226	45.32	
Oil	AX-90363747	*qProOil.4*	4	**A**	20	20.03	0.87
				G	287	19.16	
	AX-90471463	*qOilCarb.6*	6	**T**	85	20.37	1.48
				C	212	18.89	
	AX-90435033	*qOil.8*	8	**G**	34	21.25	2.19
				A	275	19.07	
	AX-90381095	*qOil.13*	13	**T**	46	19.70	0.46
				C	260	19.24	
	AX-90496790	*qProOil.16*	16	**A**	21	20.96	1.77
				G	289	19.18	
Carbohydrate	AX-90471463	*qOilCarb.6*	6	**T**	85	34.52	−1.74
				C	212	36.25	

Alleles in bold are the reference allele, while those in normal font are the alternate allele.

**Table 4:** Summary of putative genes detected in stable QTLs for the protein, oil and carbohydrate content.

QTL	Chr.	Gene ID	Position (bp)	Strand	Annotation	References
*qProOil.4*	4	*Glyma.04g003900*	318,939–321,963	F	Beta-galactosidase	[33]
		*Glyma.04g004000*	324,705–335,134	R	Hect domain ubiquitin-protein ligase	[34]
*qProOil.16*	16	*Glyma.16g207500*	36,728,513–36,732,189	F	Peroxidase	[36]
		*Glyma.16g208500*	36,806,718–36,809,299	F	Translation initiation factor 6 (eIF-6)	[37]
		*Glyma.16g208600*	36,810,473–36,813,869	F	Translation initiation factor 6 (eIF-6)	[37]
		*Glyma.16g208900*	36,838,653–36,839,376	F	Bowman-Birk serine protease inhibitor	[38]
		*Glyma.16g209300*	36,856,833–36,863,994	R	Enoyl-CoA hydratase	[39]
		*Glyma.16g209800*	36,908,675–36,915,046	R	Alpha-galactosidase	[40]
		*Glyma.16g210200*	36,933,402–36,938,940	F	hydroxyproline-rich glycoprotein	[41]
		*Glyma.16g210300*	36,943,073–36,948,828	F	Cytochrome P450 superfamily protein	[42]
*qPro.9*	9	*Glyma.09g164900*	38,902,488–38,907,721	F	Succinyl-CoA synthetase	[34]
*qPro.10*	10	*Glyma.10g078900*	8,904,408–8,904,722	R	Ribosomal protein L2	[33]
		*Glyma.10g079000*	8,945,342–8,950,202	F	ATP-dependent CLP protease	[43]
		*Glyma.10g079500*	9,145,004–9,147,592	F	Serine/threonine specific protein phosphatase	[35]
*qPro.18A*	18	*Glyma.18g072800*	6,873,417–6,878,947	R	BTB/POZ domain	[44]
*qOil.8*	8	*Glyma.08g281900*	38,660,625–38,661,689	F	AP2 domain	[7]
*qOil.13*	13	*Glyma.13g154800*	27,026,082–27,030,891	F	ABC transporter	[16]
		*Glyma.13g155600*	27,067,243–27,071,804	R	alpha/beta hydrolase	[26]
		*Glyma.13g156800*	27,201,450–27,204,098	R	Transmembrane 9 superfamily protein	[45]
*qOilCarb.6*	6	*Glyma.06g201700*	18,577,883–18,617,874	F	ATP-dependent RNA helicase	[46 ,47]
		*Glyma.06g201800*	18,631,314–18,635,564	R	CGI-140	[48]
		*Glyma.06g202000*	18,655,235–18,667,810	R	Dentin sialophosphoprotein	[46]

## Data Availability

The original contributions presented in this study are included in the article/Supplementary Material. Further inquiries can be directed to the corresponding authors.

## Supplementary Materials

The following supporting information can be downloaded at https://www.mdpi.com/article/10.3390/plants15060924/s1.
