# Supplementary material for: Genome-Wide Identification of Candidate Loci Associated with the Regulation of the Protein, Oil, and Carbohydrate Content in Soybean
Source: Plants (Basel). 2026 Mar 17;15(6):924. doi: 10.3390/plants15060924 (PMC13030290; doi:10.3390/plants15060924)
Supplement: Supplementary file 1 [file plants-15-00924-s001.zip › Supplementary Figures.pdf]

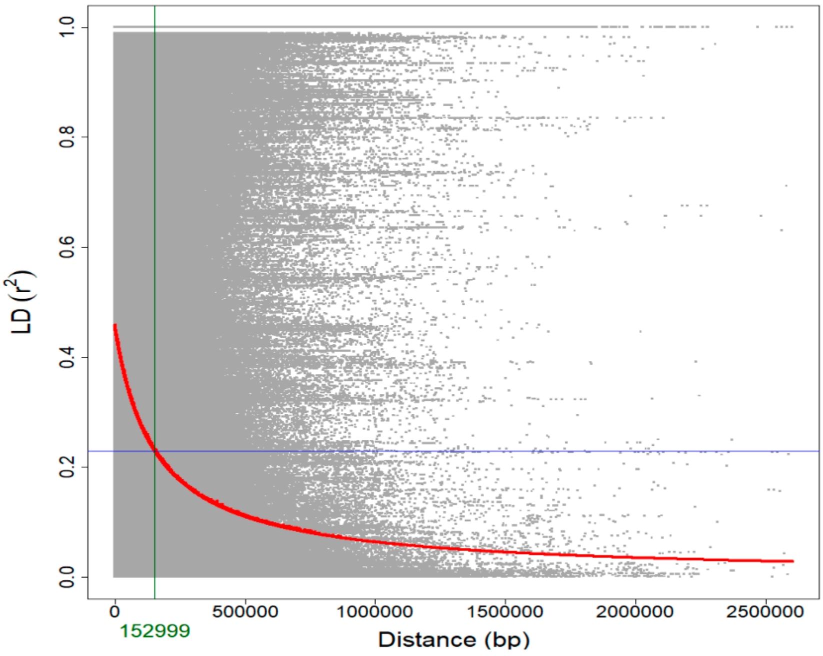


**Supplementary Figure S1.** LD decay curve displaying half decay at $r^2$ (0.22) at a physical distance of 153kb.

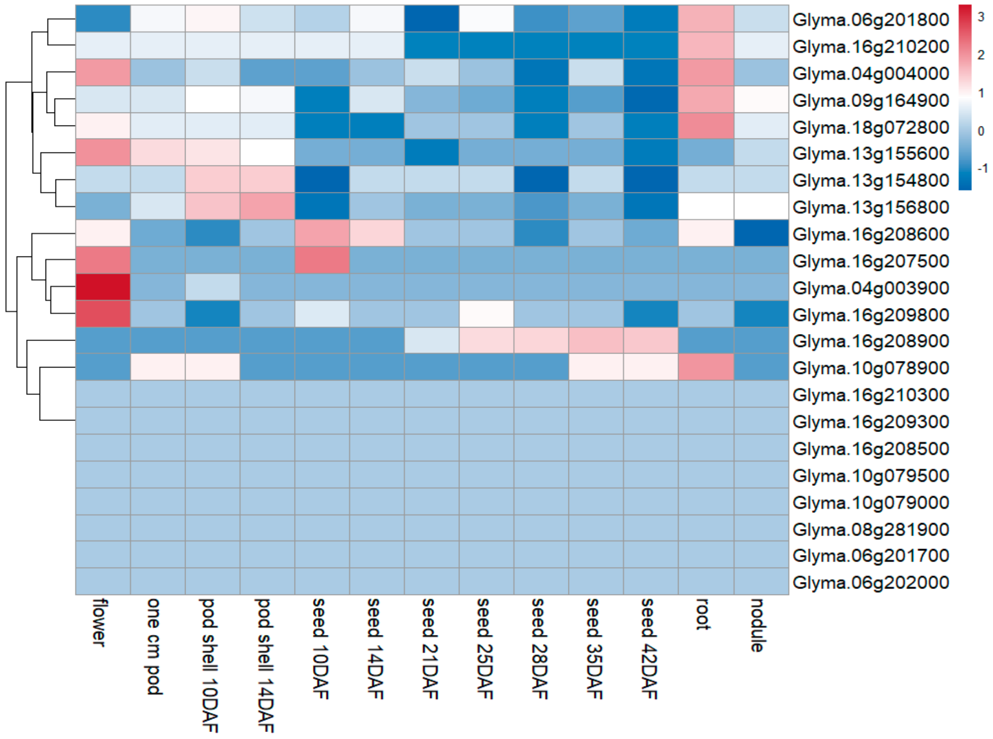


**Supplementary Figure S2.** Tissue-specific expression profiles of putative genes associated with seed protein, oil, and carbohydrate biosynthesis (Severin et al., 2010).
